# Detection Principles of Temperature Compensated Oscillators with Reactance Influence on Piezoelectric Resonator

**DOI:** 10.3390/s20030802

**Published:** 2020-02-01

**Authors:** Vojko Matko, Miro Milanovič

**Affiliations:** Faculty of Electrical Engineering and Computer Science, University of Maribor, Koroška c. 46, 2000 Maribor, Slovenia; miro.milanovic@um.si

**Keywords:** piezoelectric impedance, reactance influence on resonance, detection principle of piezoelectric oscillators

## Abstract

This review presents various ways of detection of different physical quantities based on the frequency change of oscillators using piezoelectric crystals. These are influenced by the reactance changes modifying their electrical characteristics. Reactance in series, in parallel, or a combination of reactances can impact the electrical crystal substitute model by influencing its resonant oscillation frequency. In this way, various physical quantities near resonance can be detected with great sensitivity through a small change of capacitance or inductance. A piezoelectric crystal impedance circle and the mode of frequency changing around the resonant frequency change are shown. This review also presents the influence of reactance on the piezoelectric crystal, the way in which the capacitance lost among the crystal’s electrodes is compensated, and how the mode of oscillators’ output frequency is converted to lower frequency range (1–100 kHz). Finally, the review also explains the temperature–frequency compensation of the crystals’ characteristics in oscillators that use temperature–frequency pair of crystals and the procedure of the compensation of crystals own temperature characteristics based on the method switching between the active and reference reactance. For the latter, the experimental results of the oscillator’s output frequency stability (*f*_out_ = ±0.002 ppm) at dynamical change of environment temperature (0–50 °C) are shown.

## 1. Introduction

There are many different types of oscillators using crystals as the key components of their circuit. The quartz oscillator, in particular, is uniquely suited for the manufacture of frequency selection or frequency control devices [1,2,3]. On the other hand, these oscillators can be used for the detection of various physical quantities based on the frequency change that can be triggered by mechanical influence on the crystal [4] or electrical influence on its substitute electrical model [1,5]. The purpose of this study is to provide an overview of various ways of detection of physical quantities using oscillators with a piezoelectric crystal (as an oscillating element) and reactance connected in series or in parallel. The latter influences the crystal’s electric substitute model and consequently its performance characteristics (the oscillating frequency near the resonance) [6,7,8]. The advantage of this method is great enhancement of sensor sensitivity, excellent temperature compensation, as well as compensation of capacitance among the crystal’s electrodes and other parasitic impedances.

Current state of the research field: In [9], a calibration method based on self-reciprocity is proposed for the determination of transducer sensitivity, which can be applied to both planar and focused transducers. The two-port electrical network of the experimental setup is analyzed, and a simplified measurement procedure is described in which the “impedance mismatch” problem is solved. Another paper [10] presents an equivalent circuit model, a systematic design, and an optimization method for developing a broadband annular diaphragm piezoelectric micromachined ultrasonic transducer (A-PMUT). By utilizing array analysis methods, an annular diaphragm is regarded as an array consisting of equally spaced sector diaphragms influencing each other by crosstalk effects [11]. The model successfully explains the phenomenon of multi-resonance peaks in the frequency response curve, sharing the same vibration mode. Researchers in [12] present an initial, large-scale investigation into embedding shear-mode lead zirconate titanate piezoelectric transducers into the bond line of laminate structures, near the centerline, for the ultrasonic detection of joint defects. Simulations were performed for models containing disbonds, through-thickness cracks, and voids with experimental validation of a specimen containing a void to evaluate the effectiveness of lead zirconate titanates embedded in a bondline as sensors for damage detection. In [13], implantable middle ear hearing devices were developed as a new technology to overcome the limitations of conventional hearing aids. In this article a new piezoelectric transducer based on a piezoelectric stack is proposed and designed. This new transducer, attached to the incus body with a coupling rod, stimulates the ossicular chain in response to the expansion-and-contraction of its piezoelectric stack. In [14], the research describes the design of an amplifier for a piezoelectric transducer used in underwater communication applications. The proposed architecture combines hysteretic ripple current-mode control with an adaptive soft switching technique to achieve improved power efficiency over the full output power range. This is achieved by dynamically controlling the inductor current ripple to keep the converter running in soft switching. In [5], two piezoelectric transducers were designed to meet the strict underwater application backgrounds such as high pressure, corrosion resistance, and high strength. The two models were constructed and compared with chosen sets of geometric parameters, which can also provide a reference for low-frequency transducer design. The experimental results of proposed transducers show a good consistency, which indicates that the cavity structure can reduce the resonance frequency. In [15] a general model was developed that describes the electrical responses of thickness-shear mode resonators subject to a variety of surface conditions. The model incorporates a physically diverse set of single-component loadings, including rigid solids, viscoelastic media, and fluids (Newtonian or Maxwellian). In [16] the method improving quartz oscillator temperature–frequency characteristics compensation is switching between two impedance loads. By modifying the oscillator circuit with two logic switches and two impedance loads, the oscillator can switch oscillation between two resonance frequencies. The difference in resonance frequencies compensates the temperature–frequency characteristics’ influence as well as the influence of offset and quartz crystal ageing.

This work provides an overview of ways of detection of different physical quantities using oscillators with piezoelectric crystals and load impedance connected in series or parallel through which we impact the electrical substitute crystal model and its performance characteristics. In oscillators with load capacitance or inductance in series or parallel with the crystal unit, the oscillation frequency depends on the capacitive or inductive load that is applied. The frequency will increase if the capacitive load is decreased and decrease if the load is increased. The amount of frequency change (in ppm) as a function of load capacitance is different for various ways of influencing the electrical substitute model of the piezoelectric crystal. It indicates how far from the nominal frequency (intended oscillating frequency) the resonant frequency can be forced by applying the load. In this case, it can be used for measurement purposes, allowing the measurement of various physical quantities based on capacitive and inductive influence on the quartz crystal’s oscillation frequency. A huge advantage of these ways of detection of piezoelectric oscillators is a very good temperature compensation of the crystal’s own temperature–frequency characteristics (although this is not always necessary in certain cases) and an improved impedance-to-frequency change sensitivity.

## 2. Piezoelectric Crystal Equivalent Circuit and its Impedance

**Figure 1:**
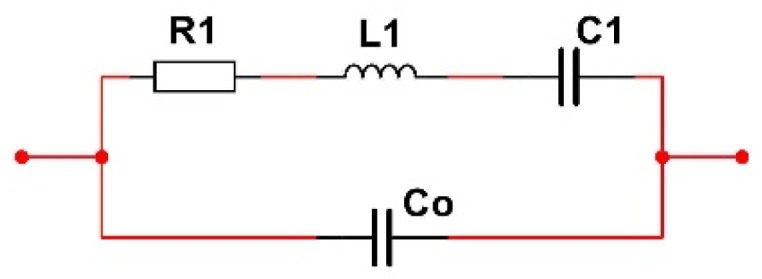
The piezoelectric crystal equivalent circuit (Butterworth Van-Dyke (BVD) model) [19,20].

The operation of a piezoelectric crystal is frequently explained using the equivalent circuit, illustrated in Figure 1, representing an electrical depiction of the quartz crystal unit [1,6,15,17,18]. The capacitance labeled C_0_ is real capacitance, comprising the capacitance between the electrodes and the stray capacitance associated with the mounting structure. It is also known as the "shunt" or "static" capacitance, and represents the crystal in a non-operational state. This can also depend on the medium where the piezoelectric is located, namely conductive or non-conductive medium. The other components represent the crystal in an operational or motional state; L_1_, C_1_, and R_1_ identify the "motional inductance", the "motional capacitance", and the "motional resistance", respectively. The motional inductance L_1_ represents the vibrating mass of the quartz plate, while the motional capacitance C_1_ represents the elasticity or stiffness of the plate. The motional resistance R_1_ represents the bulk losses occurring within the vibrating plate.

The piezoelectric crystal has two resonance frequencies, namely the series resonance frequency *f*_s_ and the parallel resonance frequency *f*_p_. The series and parallel resonance frequencies and the quality factor *Q* are specified by the following equations [1,6,21]:

(1) $$f_{s}=\frac{1}{2\pi \cdot \sqrt{L_{1}C_{1}}}$$

(2) $$f_{p}=\frac{1}{2\pi \cdot \sqrt{L_{1}\frac{C_{1}C_{0}}{C_{1}+C_{0}}}}=f_{s}\sqrt{1+\frac{C_{1}}{C_{0}}}$$

(3) $$Q=\;\frac{2\pi f_{s}L_{1}}{R_{1}}=\frac{1}{2\pi f_{s}R_{1}C_{1}}$$

The complex impedance equation for the piezoelectric crystal equivalent circuit (Figure 1) [1,6,21] is

(4) $$\underline{Z}=\frac{\left(R_{1}+j\omega L_{1}+\frac{1}{j\omega C_{1}}\right)\frac{1}{j\omega C_{0}}}{R_{1}+j\omega L_{1}+\frac{1}{j\omega C_{1}}+\frac{1}{j\omega C_{0}}}{=}^{\;}\frac{R_{1}+j\left(\omega L_{1}-\frac{1}{\omega C_{1}}\right)}{1+\frac{C_{0}}{C_{1}}-\omega^{2}L_{1}C_{0}+j\omega R_{1}C_{0}}$$

By introducing the normalized frequency Ω = *ω*/*ω*_0_, which is related to the resonance frequency $\omega_{0}=1/\sqrt{L_{1}C_{1}}$, taking into account $\omega_{0}L_{1}$= 1/$\omega_{0}C_{1}$ and $\omega_{0}$= $\omega_{s}$ and Equation (4), we can write [6,18] the following equation:

(5) $$\underline{Z}=R_{1}\frac{1+j\frac{\omega_{0}L_{1}}{R_{1}}\left(\Omega -\frac{1}{\Omega }\right)}{1+\frac{C_{0}}{C_{1}}\left(1-\Omega^{2}\right)+j\frac{C_{0}}{C_{1}}\frac{R_{1}}{\omega_{0}L_{1}}\Omega }$$

with the real part

(6) $$Re\left(\underline{Z}\right)=\frac{R_{1}}{{\left[1+\frac{C_{0}}{C_{1}}\left(1-\Omega^{2}\right)\right]}^{2}+{\left(\frac{C_{0}}{C_{1}}\frac{R_{1}}{\omega_{0}L_{1}}\Omega \right)}^{2}}$$

and the imaginary part [6,18]

(7) $$Im\left(\underline{Z}\right)=-jR_{1}\frac{C_{0}}{C_{1}}\frac{\omega_{0}L_{1}}{R_{1}\Omega }\cdot \frac{\Omega^{4}-\left[2+\frac{C_{1}}{C_{0}}-{\left(\frac{R_{1}}{\omega_{0}L_{1}}\right)}^{2}\right]\Omega^{2}+1+\frac{C_{1}}{C_{0}}}{{\left[1+\frac{C_{0}}{C_{1}}\left(1-\Omega^{2}\right)\right]}^{2}+{\left(\frac{C_{0}}{C_{1}}\frac{R_{1}}{\omega_{0}L_{1}}\Omega \right)}^{2}}$$

Taking into account various ways of oscillation of piezoelectric crystals, Table 1 shows values or value ranges for individual electrical equivalent circuit elements (Figure 1). For individual frequencies of crystal’s oscillation, it is possible to select given values for R_1_, C_1_, and L_1_.

**Figure 2:**
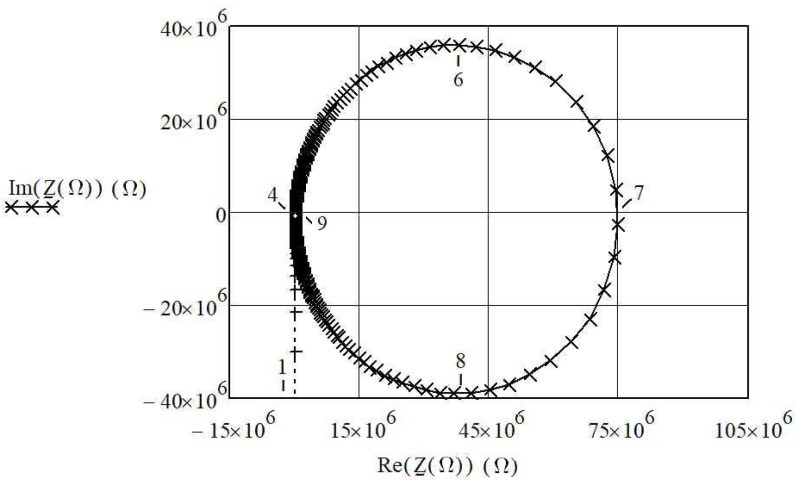
The complex impedance circle for the piezoelectric crystal equivalent circuit (C_1_ = 3.24 fF, L_1_ = 7281 H, R_1_ = 30 kΩ). Both axes are in Ohm [22].

For the selected data C_1_ = 3.24 fF, L_1_ = 7281 H, and R_1_ = 30 kΩ [6] from Table 1, a series resonance frequency *f*_s_ = 1/$(2\pi \cdot \sqrt{L_{1}C_{1})}$ = 32.768 kHz can be calculated. By introducing a normalized frequency Ω (Equations (5)–(7)), the crystal’s impedance change near the series resonance frequency (32.768 kHz) can be described in Figure 2. By changing the normalized frequency Ω from 0 to ∞, the piezoelectric crystal impedance changes (Equations (6) and (7)) in the impedance circle (Figure 2). Place 4 (Figure 2) represents series resonant frequency Ω_s_, and place 7 represents parallel resonant frequency Ω_p_. In both cases, impedance imaginary parts are zero. At Ω_s_ the impedance real value is very small, while at Ω_p_ it is very big_._ Even though the real parts of impedance differ by a factor of 2500 for the resonant frequencies Ω_s_ and Ω_p_, the resonant frequencies differ by only 0.05%. At places 6 and 8, the absolute value of the impedance imaginary parts has the greatest value.

## 3. Reactance Influence on Resonance of Piezoelectric Crystals

Different ways of detecting physical quantities based on the change in frequency of oscillators operating with piezoelectric crystals can be demonstrated by the effect of reactance on the resonant frequency of piezoelectric crystals. Presented is the influence of reactance on the piezoelectric crystal, the way in which the capacitance lost among the crystal’s electrodes is compensated, and how the mode of oscillator output frequency is converted to lower frequency range.

### 3.1. Load Capacitance Influence on Resonance

**Figure 3:**
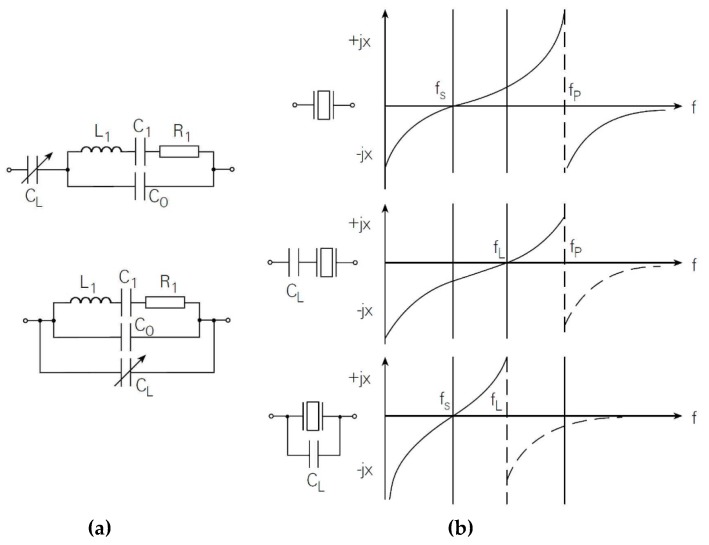
(**a**) Capacitance C_L_ influence (serial and parallel) on the piezoelectric equivalent circuit; (**b**) The reactance curves without a load capacitance C_L_, with load capacitance C_L_ in series and then in parallel with piezoelectric resonator [21].

Usually, to set the resonance frequency, the load capacitance is connected in series or in parallel with the piezoelectric crystal, depending on whether we wish to influence its series or parallel resonant frequency (Figure 3a,b) [1,3,6,21,22].

In order to calculate the influence of the series capacitance *C_L_* (Figure 3) on the series resonance frequency *f*_s_, the crystal resistance R_1_ (Equation (4)) is neglected (R_1_ = 0), so that the series impedance is obtained for the series quartz coupling and the series capacitance [6,18,23,24].

(8) $${\underline{Z}}^{*}=\frac{j\left(\omega L_{1}-\frac{1}{\omega C_{1}}\right)}{1+\frac{C_{0}}{C_{1}}-\omega^{2}L_{1}C_{0}}+\frac{1}{j\omega C_{L}}=\frac{1}{j\omega C_{L}}\frac{C_{1}+C_{0}+C_{L}-\omega^{2}L_{1}C_{1}\left(C_{0}+C_{L}\right)}{C_{0}+C_{1}-\omega^{2}L_{1}C_{1}C_{0}}$$

The new resonant frequency $f_{s}{}^{*}$ for the connection in series of both elements (crystal and *C_L_*) is

(9) $$f_{s}{}^{*}=\frac{1}{2\pi \cdot \sqrt{L_{1}C_{1}}}\sqrt{1+\frac{C_{1}}{C_{0}+C_{L}}}$$

Since in Equation (9) *C*_1_/(*C*_0_ + *C*_L_) << 1, Equation (10) can be written as

(10) $$f_{s}{}^{*}≅\frac{1}{2\pi \cdot \sqrt{L_{1}C_{1}}}\left(1+\frac{C_{1}}{2\left(C_{0}+C_{L}\right)}\right)$$

The change of the series resonant frequency Δ*f*_s_/*f*_s_ can be written as [1,6]

(11) $$\frac{\Delta f_{s}}{f_{s}}=\frac{\Delta \omega_{s}}{\omega_{s}}=\frac{\omega_{s}{}^{*}-\omega_{s}}{\omega_{s}}=\frac{C_{1}}{2\left(C_{0}+C_{L}\right)}$$

**Figure 4:**
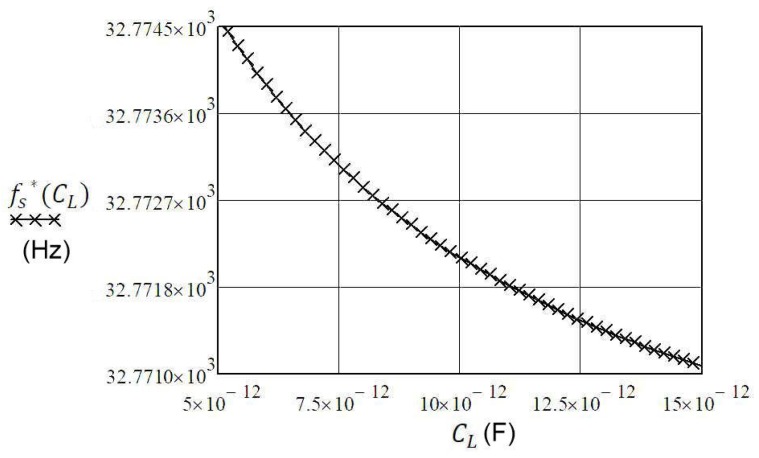
The range of resonant frequency changing $f_{s}{}^{*}$ = 32.7710 to 32.7745 kHz due to the changing of capacitance C_L_ in the range of 5–15 pF [18,21].

If we know the range of capacitance *C_L_* change, Equation (10) can reflect the change of frequency $f_{s}{}^{*}$ (for example, the capacitance change *C_L_* = 5–15 pF (Figure 4)). For greater changes of capacitance *C_L_* > 15 pF, the frequency change $f_{s}{}^{*}$ is a lot smaller.

(12) $$f_{s}^{*}\left(C_{L}\right)=\frac{1}{2\pi \cdot \sqrt{L_{1}C_{1}}}\left(1+\frac{C_{1}}{2\left(C_{0}+C_{L}\right)}\right)$$

### 3.2. Capacitance Compensation of Piezoelectric Electrodes

**Figure 5:**
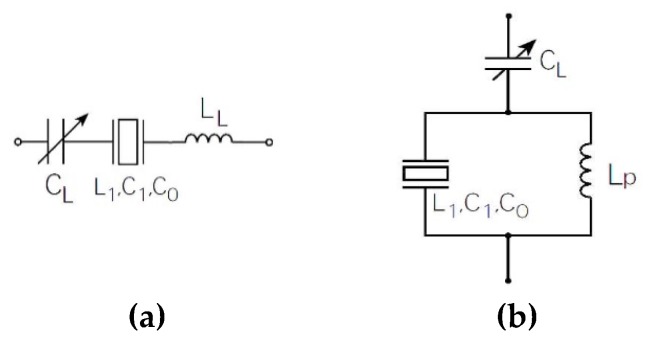
(**a**) Compensation C_0_ with the inductance L_L_ connected in series; (**b**) compensation C_0_ with parallel inductance L_p_.

Capacitance C_0_ (Figure 1) represents the capacitance between the electrodes of the piezoelectric crystal, and can be compensated with the inductance L_L_ in series with the crystal (Figure 5a) provided that (Equation (13)) [6,18,20,25] is

(13) $$L_{L}=\frac{1}{\omega_{s}^{2}C_{0}}$$

**Figure 6:**
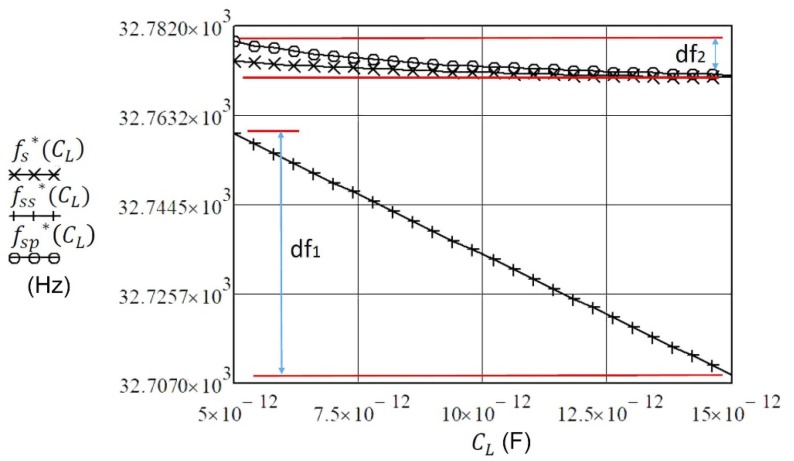
A comparison between the frequency changes $f_{s}{}^{*}\left(C_{L}\right)$ (Equation (12)), frequency $f_{ss}{}^{*}\left(C_{L}\right)$ (Equation (14)), and $f_{sp}{}^{*}\left(C_{L}\right)$ (Equation (16)) as result of changing of capacitance C_L_ (in the range from od 5–15 pF).

If we take into account Equation (9) and Equation (13) (compensation C_0_), the new resonant frequency ($f_{ss}{}^{*}$) is expressed with Equation (14). With this equation, enhanced pulling sensitivity *df*_1_ near the series resonant frequency *f*_s_ (Equation (1)) is achieved, as shown in Figure 6.

(14) $$f_{ss}{}^{*}\left(C_{L}\right)≅\frac{1}{2\pi \cdot \sqrt{L_{1}C_{1}}}\left(1+\frac{C_{1}}{2\left(C_{0}-\frac{1}{\omega_{s}^{2}L_{L}-{\frac{1}{C}}_{L}}\right)}\right)$$

Conversely, with high load capacitance C_L_ the pulling sensitivity is very small, so 100 pF likely represents, approximately, the upper limit.

The pulling sensitivity *df*_2_ (Figure 6) of the crystal unit can be also increased if the C_0_ is compensated by parallel inductance *L_p_* to the crystal (Figure 5b). The equation for compensation is

(15) $$L_{p}=\frac{1}{\omega_{s}^{2}C_{0}}$$

(16) $$f_{sp}{}^{*}\left(C_{L}\right)=\frac{1}{2\pi \cdot \sqrt{L_{1}C_{1}}}\sqrt{1+\frac{C_{1}}{C_{L}}}$$

When *C_L_* is connected in series with the piezoelectric crystal (without compensation C_0_), the frequency change $f_{s}{}^{*}\left(C_{L}\right)$ is the smallest in the range 5–15 pF (Figure 6). With connection *C_L_* in parallel and compensation C_0_, the change of frequency $f_{sp}{}^{*}\left(C_{L}\right)$ is somewhat greater but nonlinear.

With connection in series C_L_ and compensation C_0_, the change of frequency $f_{ss}{}^{*}\left(C_{L}\right)$ is the greatest (the greatest frequency pulling) and linear near the resonant frequency *f*_s_. This sensitivity and linearization study is described in more detail and compared with other studies in [26].

### 3.3. Load Inductance Influence on Resonance

**Figure 7:**
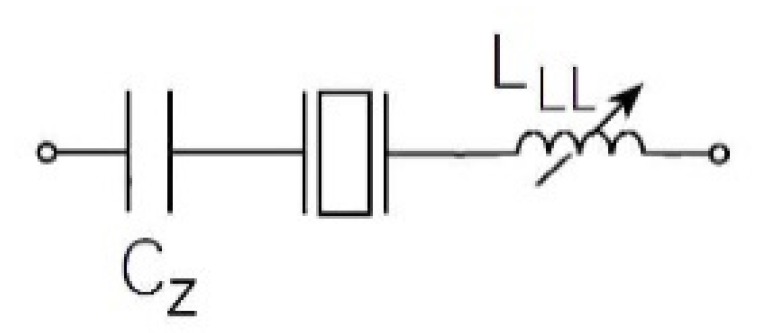
Load inductance L_LL_ in series with the piezoelectric crystal.

There is another possibility to increase the pulling sensitivity and this is to replace the series load capacitor C_L_ with a series load inductance L_LL_ (Figure 7) [6,18,25].

Capacitor C_Z_ (Figure 7) is intended to cancel the effective series resistance of the circuit and not to act as a load capacitor for the crystal. Capacitor C_Z_ = ~10 nF and plays the role of connecting capacitor that has practically no influence on the piezoelectric series resonant frequency *f*_s_, which is why in Equation (14) the expression 1/C_L_ can be ignored and left out, which gives us Equation (17) [26].

(17) $$f_{sss}{}^{*}\left(L_{LL}\right)≅\frac{1}{2\pi \cdot \sqrt{L_{1}C_{1}}}\left(1+\frac{C_{1}}{2\left(C_{0}-\frac{1}{\omega_{s}^{2}L_{LL}}\right)}\right)$$

**Figure 8:**
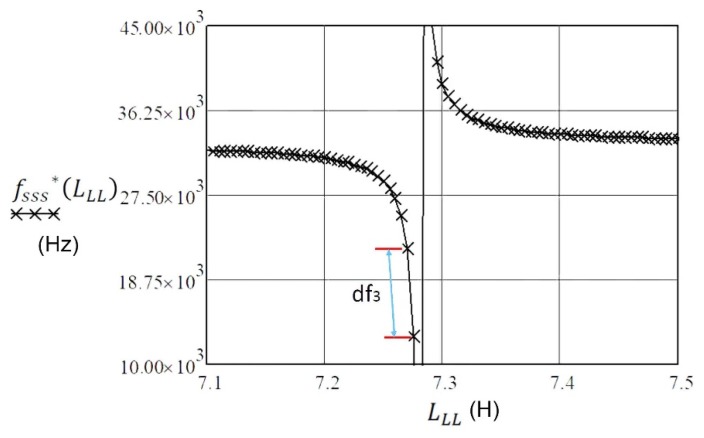
Inductance frequency pulling for data: C_1_ = 3.24 fF, L_1_ = 7281 H, R_1_ = 30 kΩ, *f*_0_ = 32.768 kHz, and for inductance L_LL_ change in the range 7.1–7.5 H.

If we take in account (experimental data) that C_1_ = 3.24 fF, L_1_ = 7281 H, R_1_ = 30 kΩ, and *f*_0_ = 32.768 kHz from Table 1, and that inductance L_LL_ is changed in the range 7.1–7.5 H in Equation (17), we can show the frequency pulling *df*_3_, which is almost linear in Figure 8 and for the experimental data amounts to 8.75 kHz/5 mH. There is, however, one potential disadvantage to this method. The effective Q of the combination L_1_ and L_LL_ (Figure 7) is dramatically reduced due to the relatively low Q of the inductance L_LL_ employed. Furthermore, there is also an additionally tuned circuit created, consisting of L_LL_, C_1_ and C_0_ of the piezoelectric element.

All piezoelectric crystals have spurious resonances (unwanted resonance responses) beside the main resonance frequency. They are represented in the equivalent circuit by additional resonant circuits in parallel with R_1_, L_1_, and C_1_ [3], and they are just other vibration modes at higher frequency values.

### 3.4. Ceramic Resonators

A cheaper alternative to the quartz crystal is a ceramic resonator. This device uses mechanical resonance of a piezoelectric ceramic, typically lead zirconate titanate, which vibrates in various mechanical modes depending on the chosen resonant frequency *f*_s_cer_. The approximate frequency ranges are shown in Table 2 [27,28,29,30].

The equivalent circuit of the ceramic resonator is identical to that of the quartz crystal (Figure 1), but the component values give it orders of magnitude lower Q. In terms of oscillation frequency accuracy, the resonator sits between the quartz crystal and the LC resonant circuit (an electric circuit consisting of an inductor, represented by the letter L, and a capacitor, represented by the letter C, connected together). The temperature compensation of a resonator is of the order of 10^−5^/°C compared to the more than 1 ppm/°C achievable with quartz, and the 10^−3^–10^−4^/°C of LC circuits. Its initial frequency tolerance is of the order of ±0.5%, whereas quartz routinely achieves ±0.003%. To achieve these figures using LC, circuits would need a trimming adjustment. On the other hand, the resonator is cheaper and smaller than the quartz crystals and can use the same or similar oscillator circuits. An advantage of the resonator is that because of its lower Q, the oscillation will start up more quickly than for an equivalent crystal circuit, which makes it attractive for applications that spend a lot of their time in “sleep” mode with the oscillator powered off. All of these characteristics make the ceramic resonator the component of choice for frequency control of low- and mid-performance digital products, where a stable clock frequency is needed but where absolute accuracy or close control of temperature compensation is not a requirement. It is available in a wide range of standard frequencies (Table 2), matched to particular consumer applications [27,28,29,30,31].

### 3.5. Oscillator Frequency Transformation Using Reference Oscillator

**Figure 9:**
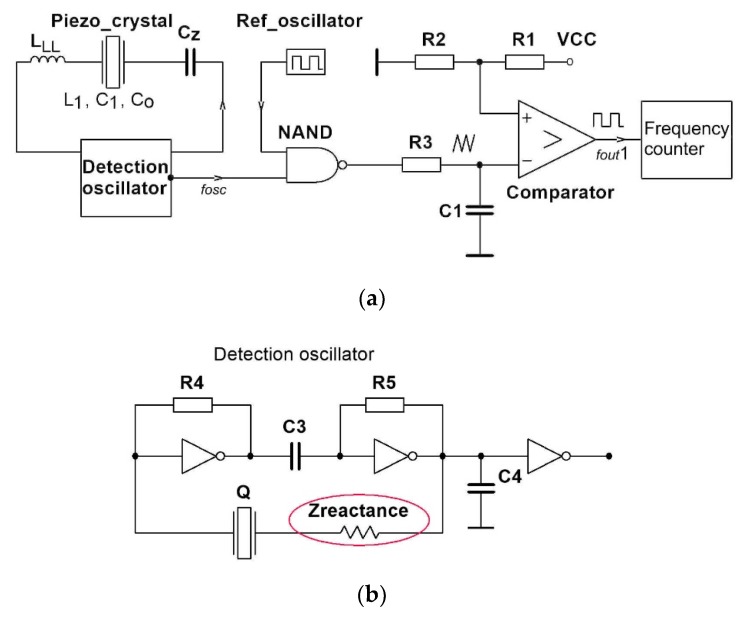
(**a**) Higher oscillator frequency (*f*_osc_) signal transformation to lower frequency range (*f*_out1_ = 1–100 kHz), (NAND stands for digital AND gate with negated output, VCC = 5 V); (**b**) detection oscillator.

Oscillator frequency measurement by frequency counter is more accurate when the frequency *f*_osc_ of detection oscillator (Figure 9a) >1 MHz) is transformed to lower frequency range (*f*_out1_ = 1–100 kHz). For this transformation, a reference oscillator and a low pass filter are used and a precise comparator circuit (or Schmitt trigger circuit) is added, which transforms a triangular signal to a rectangular signal. When using this transformation, more decimal places on the display of the frequency counter (experimentally used HM8123-X, Hameg Instruments) are accurate [1,2,31].

The output frequency *f*_out1_ (Figure 9a) is defined as

(18) $$f_{out1}=\left[\left(f_{ref}+df_{ref}\left(T\right)+df_{ref}\left(t\right))-(f_{osc}+df_{osc}\left(T\right)+df_{osc}\left(t\right)\right)\right]+df_{c\_error1}$$

If crystal ageing $df_{osc}\left(t\right)$ and reference oscillator crystal ageing $df_{ref}\left(t\right)$ (*t* *−* time) are approximately the same (they compensate), we get Equation (19), where the temperature–frequency change ($df_{ref}\left(T\right),\;df_{osc}\left(T\right))$ (T—temperature) is not approximately the same. In Equation (19) the frequency measurement error $df_{c\_error}$ (counter error) also has to be taken into account, which includes also

(19) $$f_{out1}=\left[\left(f_{ref}+df_{ref}\left(T\right))-(f_{osc}+df_{osc}\left(T\right)\right)\right]+df_{c\_error1}v$$

**Figure 10:**
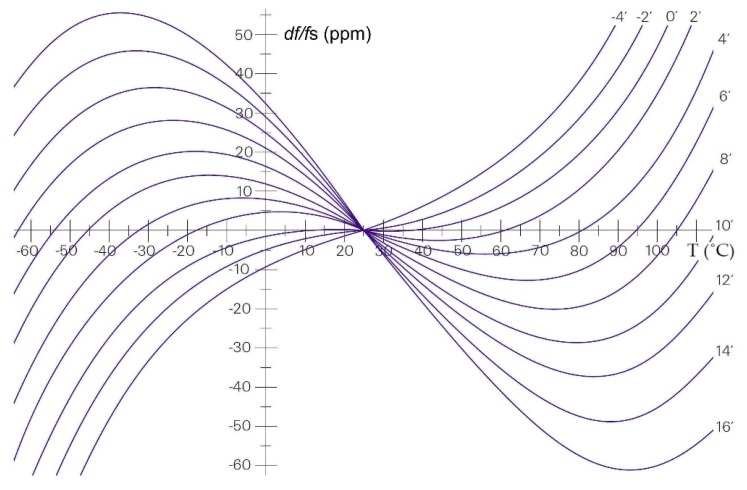
Temperature–frequency characteristics for thickness-shear mode AT-cut crystals with angle of cut from −4′ to +16′ as a parameter [6,21,32,35].

**Figure 11:**
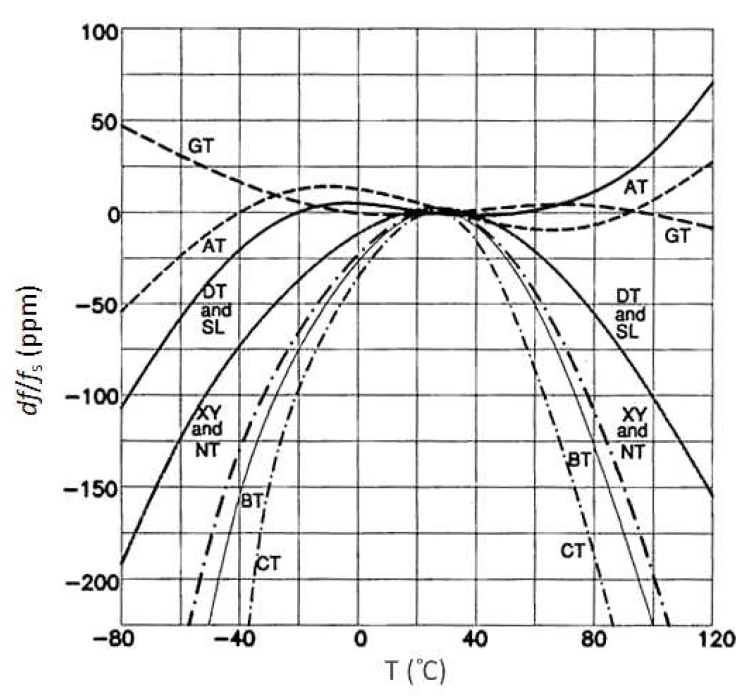
A temperature sensitivity of the synthetic crystals dependent on cutting angle [6,21,32,35].

A piezoelectric crystal (Figure 9a) must have the temperature–frequency characteristics as much as possible independent of the temperature (AT-cut 0′ (the crystal’s x axis is inclined by 35°15′ from the z (optic) axis, typically operate in fundamental mode at 1–30 MHz, Figure 10 and Figure 11) in the working temperature range of 10–40 °C. The reference oscillator (Figure 9a) is an oven-controlled crystal oscillator (OCXO). Experimental data were: OCT18T5, 1.25–100.0 MHz, stability 0–60 °C = ±0.01 ppm, ageing ±0.5 ppm/year, supply voltage variation ±5%. Using the method shown in Figure 9, stability of *f*_out1_ = ±2 ppm can be achieved in the temperature range of 10–40 °C (Figure 10, cutting angle 0′) [3].

## 4. Temperature–Frequency Characteristics Compensation of Piezoelectric Crystals

With accurate detection principles using piezoelectric oscillators, the temperature stability of piezoelectric crystals is important. Different methods of temperature–frequency compensation of crystals in oscillators using temperature–frequency pairs of crystals and a procedure for compensation of the inherent temperature–frequency characteristics of crystals based on the method of switching between active and reference reactances are shown. For the latter, experimental results of the dynamic stability of the oscillator output frequency are also presented.

### 4.1. Crystal Temperature Sensitivity

Due to the crystal’s physical properties, AT-cut crystals are predominantly used in oscillator circuits [1,32]. Their main advantage is the low temperature sensitivity in the temperature range between 10 and 40 °C. The curves are represented as the cubical parabola with the temperature intersection point of 25 °C, depending on the crystal cut angle and the mechanical construction (Figure 10) [1,33,34]. Equation (18) describes the piezoelectric crystal’s temperature–frequency change (in ppm) with regard to the temperature change in the range of −60 to +110 °C (Figure 10).

(20) $$\frac{df}{f}=A\left(T-T_{ref}\right)+B{\left(T-T_{ref}\right)}^{2}+C{\left(T-T_{ref}\right)}^{3}$$

where *T* is environment temperature; *T_ref_* is reference temperature; and A, B, and C are coefficients determined with regard to the respective angles of the cut [6,21,35].

Figure 11 demonstrates the typical synthetic crystal temperature–frequency characteristics in case of differently cut crystals [21]. The resonator plate can be cut from the source crystal in many different ways. At the CT-cut of the crystal (300–900 kHz, face shear, 38°) the temperature–frequency curve is a downward parabola. At the GT-cut of the crystal (0.1–3 MHz, width-extensional, 51°7’) the temperature coefficient between −25 to +75 °C is near-zero, due to cancelling effect between two modes. At the BT-cut (0.5–200 MHz, thickness shear, −49°8’), the characteristic is similar to the AT-cut operating in b-mode (fast quasi-shear). It has well known and repeatable characteristics. DT-cut (75–800 kHz, face shear, −52°) is similar to CT-cut. SL-cut is (−57°) a face-shear. The temperature–frequency curve is a downward parabola. The temperature coefficient is lower than the CT-cut, where the frequency range permits. At the XY-cut (3–85 kHz, length–width flexure) the crystal is smaller than other low-frequency cuts, and has low impedance and low C_0_/C_1_ ratio. The chief application is the 32.768 kHz crystal. NT-cut (8–130 kHz) is length–width flexure (bending) [6,17,35].

### 4.2. Piezoelectric Crystal Temperature–Frequency Characteristics Compensation

In case of temperature-dependent piezoelectric crystals and an external influence on the oscillating frequency with an additionally connected capacitance or inductance, the crystal oscillation cannot be temperature-compensated in the temperature range −20 to +70 °C in the same way as it is possible to do with TCXO or OCXO oscillators that are hermetically closed in the housing. For this reason, two ways of compensation were used. In the first case, two oscillators and two crystals with approximately the same temperature characteristics (temperature pairs) were used. In the second case, one oscillator and one crystal were used, and the switching method between two impedances was employed.

#### 4.2.1. Similar Crystal Temperature–Frequency Characteristics Compensation

**Figure 12:**
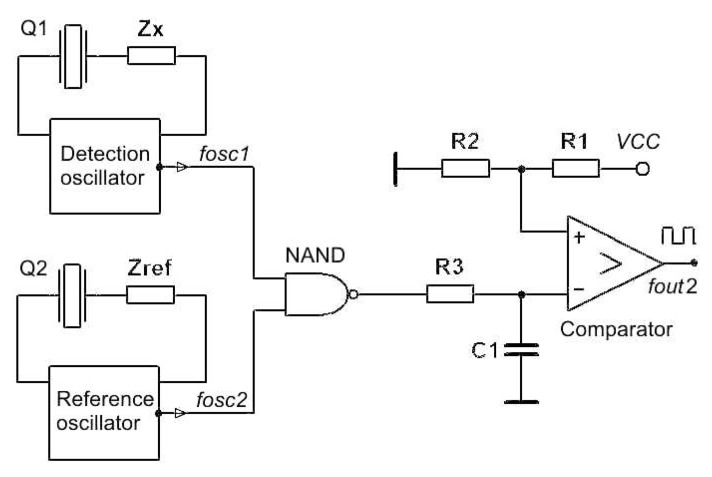
Detection principle by two oscillators with similar crystal temperature–frequency characteristics.

In the case of two crystals of similar temperature–frequency characteristics, the two oscillators and two crystals are approximately the same frequency (Figure 12) [1,2,31,36,37]. Impedance Z_ref_ is the same as Z_x_ and serves to create the same oscillating conditions in terms of impedance between the piezoelectric crystals. The frequency difference between both oscillators is ≅ 1 kHz.

The changing of impedance Z_x_ triggers the change of the oscillator’s frequency *f*_osc1_ in the range between 1 and 100 kHz, where at the low pass filter output (R_3_, C_1_) a triangular signal is created that is changed, using a comparator, into the square one that is more suitable for a frequency measurement of greater quality [1,2,38,39]. Equation (21) describes the output frequency *f*_out2_ at which the crystal ageing can be ignored, since it is the same for the two crystals, as described in Equations (18) and (19).

(21) $$f_{out2}=\left[\left(f_{osc1}+df_{osc1}\left(T\right))-(f_{osc2}+df_{osc2}\left(T\right)\right)\right]+df_{c\_error2}$$

Temperature–frequency changes ($df_{osc1}\left(T\right)\;and\;df_{osc2}\left(T\right))$ in Equation (21) are not equal, and they are not fully compensated, because the temperature–frequency characteristics of the quartzes Q1 and Q2 are very similar but not equal. The actual stability of the output frequency f_out2_ is ±1 ppm in the temperature range 10–40 °C (Figure 10, cutting angle 0′) [3,6].

#### 4.2.2. Crystal’s Temperature–Frequency Characteristics Compensation by Switching between the Load Capacitances

**Figure 13:**
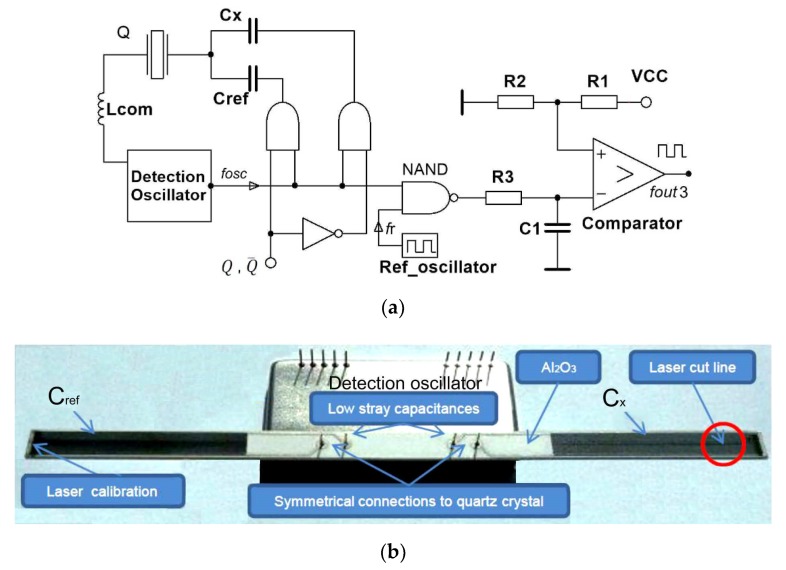
(**a**) Detection principle by one oscillator and the switching mode method compensating crystal’s own temperature–frequency characteristics and crystal’s electrode capacitance; (**b**) experimental piezoelectric detection oscillator with two equal capacitances C_x_ and C_ref_ and oscillator symmetrical construction [16].

Figure 13 shows the switching method for the compensation of the crystal’s own temperature–frequency characteristics by two capacitances C_ref_ and C_x_. Simultaneously, inductance L_com_ compensates capacitance C_0_ between the piezoelectric crystal electrodes, which improves the pulling sensitivity. The measurement method is based on the oscillator and the switching part of the circuit, alternatively switching reactances with the digital signal (Q=1, $\bar{Q}=0$) [1,2,16,22,31,40,41]. The output frequency *f*_out3_ (Equation (22) represents the output oscillator frequency, which is synchronously measured with regard to the switch of the digital signal. The switch time duration is in the range millisecond to seconds.

(22) $$f_{out3}\left(Q,\bar{Q}\right)=\left[\begin{array}{c} \left\lceil (f_{Cref}\left(t_{1}\right)+df_{Cref}\left(T_{1}\right)+df_{Cref}\left(t_{1}\right)-f_{r})+f_{c\_errorQt1}\right\rceil \\ -\lceil (f_{Cx}\left(t_{2}\right)+df_{Cx}\left(T_{2}\right)+df_{Cx}\left(t_{2}\right)-f_{r})+f_{c\_error\bar{Qt2}}\rceil \end{array}\right]$$

(23) $$f_{out3}\left(Q,\bar{Q}\right)=\left[\left(f_{Cref}\left(t_{1}\right)-f_{Cx}\left(t_{2}\right))+(f_{c\_errorQt1}-f_{c\_error\bar{Qt2}}\right)\right]$$

When the capacitances C_x_ and C_ref_ are the same, *f*_osc_ remains the same at Q=1 and $\bar{Q}=0$ and depends on the crystal resonant frequency *f*_s_ (Equation (1)), crystal temperature characteristics *f*_s_(T), and its ageing *f*_s_(t). However, when the capacitances are different, the frequency *f*_osc_ depends on the quartz crystal resonant frequency *f*_s_, the dC_x_ change (frequency pulling) and crystal temperature characteristics *f*_s_(T), and its ageing *f*_s_(t). In case of the difference of both frequencies f_osc_ for Q=1 and $\bar{Q}=0$, d*f*(T) and d*f*(t) compensate because only one temperature–frequency quartz characteristic is involved. In Equation (22), the crystal’s ageing $df_{Cref}\left(t_{1}\right)$ and $df_{Cx}\left(t_{2}\right)$ (*t*—time) are approximately the same and they compensate each other. Temperature–frequency changes $df_{Cref}\left(T_{1}\right)$ and $df_{Cx}\left(T_{2}\right)$ (T−temperature) are the same and they compensate each other when the temperature change is not so high in a short period of time up to 1 second. Reference frequency *f*_r_ from the reference oscillator is also compensated in Equation (22) by two switches, namely $Q\;\mathrm{and}\;\bar{Q}$. We get Equation (23), where the frequency measurement counter error $f_{c\_errorQt1}-f_{c\_error\bar{Qt2}}$ is subtracted and reduced to minimum. This method highly compensates the quartz crystal’s temperature–frequency characteristics. The stability of the output frequency f_out3_ is ±0.001 ppm in the temperature range 10–40 °C (Figure 10, cutting angle 0′) [3,16,18].

#### 4.2.3. Crystal’s Own Temperature–Frequency Characteristics and Simultaneous Crystal Electrode Capacitance Compensation with one Inductance

**Figure 14:**
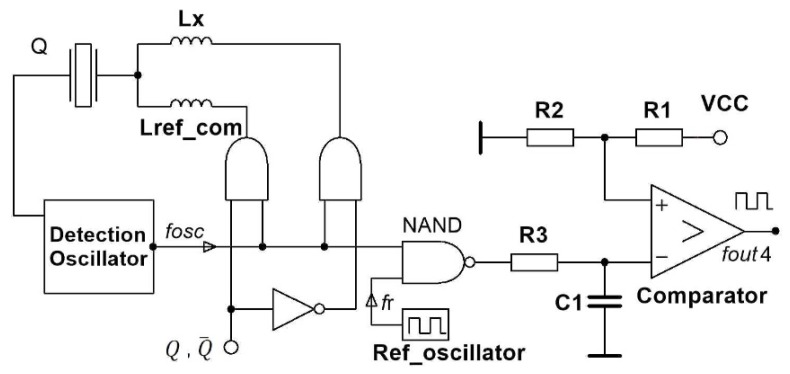
Detection principle by one oscillator and switching mode method compensating the crystal’s temperature–frequency characteristics and the crystal’s electrode capacitance by the same inductance.

Figure 14 shows the switching mode method for the compensation of the crystal’s own temperature–frequency characteristics by two inductances, L_ref_com_ and L_x_ (L_ref_com_ ≅ L_x_) [1,2,16,22,31,40,41]. Capacitance C_0_ compensation between the piezoelectric crystal’s electrodes is achieved by the value of L_ref_ and L_x_, and by the already explained Equations (13) and (14). The measurement method is similar as described in Section 4.2.2.

The output frequency *f*_out4_ (Figure 14) can be written with Equation (24), in which the crystal’s ageing $df_{Lref}\left(t_{1}\right)$ and $df_{Lx}\left(t_{2}\right)$ are approximately the same and they compensate each other. Temperature–frequency changes $df_{Lref}\left(T_{1}\right)$ and $df_{Lx}\left(T_{2}\right)$ are almost the same and they compensate each other, when the temperature change is not so high in a short period of time. Reference frequency *f*_r_ from reference oscillator is also compensated in Equation (24) by two switches, $Q,\bar{Q}$.We get Equations (25), where the frequency measurement counter error $f_{c\_errorQt1}-f_{c\_error\bar{Qt2}}$ is subtracted and reduced to minimum. This method highly compensates the quartz crystal’s temperature–frequency characteristics.

(24) $$f_{out4}\left(Q,\bar{Q}\right)=\left[\begin{array}{c} \left\lceil (f_{Lref}\left(t_{1}\right)+df_{Lref}\left(T_{1}\right)+df_{Lref}\left(t_{1}\right)-f_{r})+f_{c\_errorQt1}\right\rceil \\ -\lceil ((f_{Lx}\left(t_{2}\right)+df_{Lx}\left(T_{2}\right)+df_{Lx}\left(t_{2}\right)-f_{r}+f_{c\_error\bar{Qt2}})\rceil \end{array}\right]$$

(25) $$f_{out4}\left(Q,\bar{Q},t\right)=\left[\left(f_{Lref}\left(t_{1}\right)-f_{Lx}\left(t_{2}\right))+(f_{c\_errorQt1}-f_{c\_error\bar{Qt2}}\right)\right]$$

**Figure 15:**
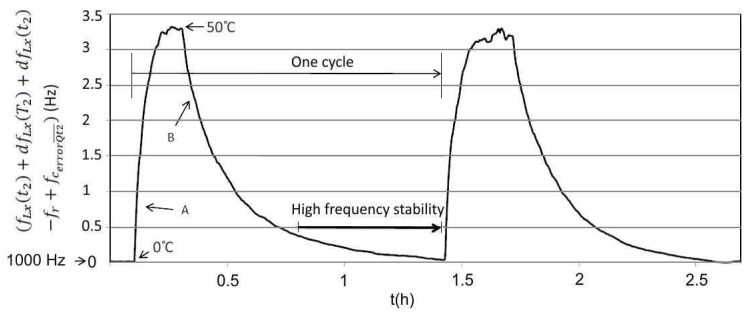
Frequency stability $\left((f_{Lx}\left(t_{2}\right)+df_{Lx}\left(T_{2}\right)+df_{Lx}\left(t_{2}\right)-f_{r}+f_{c\_error\bar{Qt2}}\right)$ occurring when changing the temperature in the range 0–50 °C (measurement time 2.5 h—two cycles).

Figure 15 shows experimental results of the frequency stability $\left(f_{Lx}\left(t_{2}\right)+df_{Lx}\left(T_{2}\right)+df_{Lx}\left(t_{2}\right)-f_{r}+f_{c\_error\bar{Qt2}}\right)$ (Equation (24) (at the beginning of temperature cycling the stable frequency $f_{out4}\left(\bar{Q}\right)$ was 1000 Hz at 0 °C) occurring when the temperature is changed in the range of 0–50 °C and fixed value L_x_ (Figure 14). The crystal used (5 MHz) in the experiment was an AT-cut (cut angle: 0′) quartz crystal with temperature change ±5 ppm in the range 10–40 °C. The A and B areas (Figure 15) illustrate dynamic change of frequency at the temperature change ranging from 0 to 50 °C and back to 0 °C.

**Figure 16:**
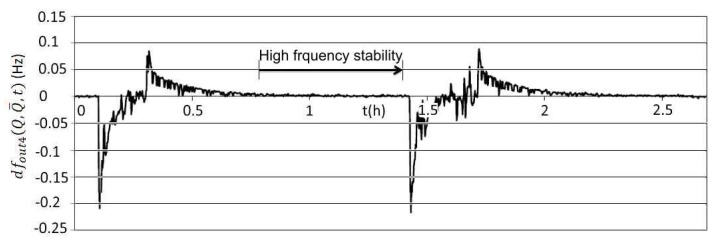
Frequency difference stability $df_{out4}\left(Q,\bar{Q},t\right)$ at the change of temperature in the range 0–50 °C, and fixed value L_x_ (measurement time 2.5 h—two cycles).

Figure 16 illustrates frequency stability $df_{out4}\left(Q,\bar{Q},t\right)$ (Equation (25)) during the change of temperature in the range 0–50 °C at the fixed value L_x_ (Figure 14) once both frequencies are deducted. Deduction of both frequencies in relation to $Q\;\mathrm{and}\;\bar{Q}$ signals is performed by LabVIEW software. In addition, Figure 16 also illustrates the temperature compensation of the quartz crystal natural temperature characteristics because only one characteristics of the crystal is involved.

The comparison of results in Figure 15 and Figure 16 points to dynamic frequency stability change (Figure 16) and to the high frequency difference stability (±0.01 Hz) area when the temperature changes less quickly. The stability of the output frequency *f*_out3_ is ±0.002 ppm in the temperature range 10–40 °C (Figure 10, cutting angle 0′) [3,16,18].

## 5. Discussion

The impedance characteristics as well as series or parallel resonant frequency of the piezoelectric crystals near the resonance can be changed with reactance. The impedance conditions can be best demonstrated by introducing the norm frequency Ω, where the real impedance is the smallest at series resonant frequency and the greatest at the parallel resonant frequency. The imaginary part of impedance in both cases is zero. While the serial load capacitance influences series resonant frequency, the parallel load capacitance influences the parallel resonant frequency. Based on this influence the pulling sensitivity is determined. The latter can be further enhanced by compensating the crystal’s capacitance C_0._ The comparison of characteristics from Figure 6 shows that the pulling sensitivity is the greatest and linear (d*f*_1_) near the piezoelectric crystal series resonance frequency at the compensation of capacitance C_0_. The serial load inductance can also increase the pulling sensitivity so that we calculate the serial load inductance L_LL_ for the compensation C_0_. Close to the value L_LL,_ the pulling sensitivity (d*f*_3_) is increased when L_LL_ is changed (Figure 8).

Since the ceramic piezo resonators have the same substitute equivalent circuits of quartz crystals, they can be treated in the similar way when they operate in the oscillating mode. Additionally, the oscillating circuits are similar or the same as in quartz crystals. Due to the lower Q-factor, they have a quicker “start-up” in oscillators.

When measuring the frequency with the help of a reference oscillator OCXO and the transformation of the signal into a square one, the measurement is more accurate. This is because the frequencies greater than 1 MHz are transformed into the range 1–100 kHz (at lower frequencies the frequency counters measure frequency more precisely for several decimal points as they do at the frequencies higher that 1 MHz).

Temperature dependence of piezoelectric crystals is present in all cuts and is expressed in ppm. For a precise measurement when using oscillators and piezoelectric crystals and outside reactances, the compensation of piezoelectric crystal’s own temperature–frequency characteristics is advisable. When using two oscillators (Figure 12) and temperature pair of crystals that have as similar characteristics as possible, this compensation is in the range below 1 ppm. However, when we use one oscillator, one crystal and the switching of two reactances this compensation (crystal’s own temperature–frequency compensation) is approximately 0.001 ppm [16]. This method and the results were confirmed by the experimentally produced prototype (Figure 13b).

The factors affecting frequency stability such as wide operating temperature range, ageing and drive level, as well as all other crystal characteristics influencing the stability should also be considered because a stable oscillator circuit plays an important role in the frequency pulling sensitivity increase [32]. Frequency stability also depends on the temperature coefficient of the compensation inductance L_com_ and L_ref_com_ material. Stability of the electronic circuit depends upon the circuit type and quality of its elements. It is also important that the drive level of the quartz crystal does not exceed 5 μW [34].

## 6. Conclusions

This review presents various ways of detection with piezoelectric crystals in oscillator circuits with additionally integrated outside reactances and temperature compensations of the crystal’s own temperature–frequency characteristics for measurement purposes. The review provides possible oscillator’s frequency, sensitivity and linearity settings. The increased pulling range can be used for the determination of many different measurements such as low capacitance change, nano-inductance, nano-extension and compression, nano-positioning, angle, pressure, nano-force, and other non-electrical quantities.

## Figures and Tables

**Table 1:** Element data for electrical substitute model of the piezoelectric crystal (quartz) (Figure 1) for various frequencies of oscillation (Equation (7)) [6].

	*f*_S_	R_1_	C_1_	L_1_
Bending vibration	1–50 kHz	5–50 kΩ	0.01 pF	10^4^ –10^3^ H
Longitudinal vibration	50–200 kHz	2–5 kΩ	0.10 pF	10–100 H
Surface vibration	150–800 kHz	0.5–10 kΩ	0.02 pF	1–10 H
Thickness shear vibration	0.5–20 MHz	2–2000 Ω	0.01 pF	10–100 mH

**Table 2:** Frequency ranges for various mechanical modes vibration [30].

	*f*_s_cer_
Longitudinal mode	30 kHz–1 MHz
Area mode	100 kHz–2 MHz
Thickness shear mode	1 MHz–10 MHz
Expansion thickness mode	2 MHz–100 MHz
Surface wave mode	10 MHz–1 GHz
